# Repetitive RNA unwinding by RNA helicase A facilitates RNA annealing

**DOI:** 10.1093/nar/gku523

**Published:** 2014-06-09

**Authors:** Hye Ran Koh, Li Xing, Lawrence Kleiman, Sua Myong

**Affiliations:** 1 Department of Physics, University of Illinois, Urbana, IL 61801, USA; 2 Institute for Genomic Biology, University of Illinois, Urbana, IL 61801, USA; 3 Lady Davis Institute for Medical Research and McGill AIDS Centre, Jewish General Hospital, Montreal, Quebec, H3T 1E2, Canada; 4 Department of Medicine, McGill University, Montreal, Quebec, H3G 1Y6, Canada; 5 Department of Bioengineering, University of Illinois, Urbana, IL 61801, USA; 6 Physics Frontier Center (Center of Physics for Living Cells), University of Illinois, Urbana, IL 61801, USA; 7 Biophysics and Computational Biology, 1110 W. Green St., Urbana, IL 61801, USA

## Abstract

Helicases contribute to diverse biological processes including replication, transcription and translation. Recent reports suggest that unwinding of some helicases display repetitive activity, yet the functional role of the repetitiveness requires further investigation. Using single-molecule fluorescence assays, we elucidated a unique unwinding mechanism of RNA helicase A (RHA) that entails discrete substeps consisting of *binding*, *activation*, *unwinding*, *stalling* and *reactivation* stages. This multi-step process is repeated many times by a single RHA molecule without dissociation, resulting in repetitive unwinding/rewinding cycles. Our kinetic and mutational analysis indicates that the two double stand RNA binding domains at the N-terminus of RHA are responsible for such repetitive unwinding behavior in addition to providing an increased binding affinity to RNA. Further, the repetitive unwinding induces an efficient annealing of a complementary RNA by making the unwound strand more accessible. The complex and unusual mechanism displayed by RHA may help in explaining how the repetitive unwinding of helicases contributes to their biological functions.

## INTRODUCTION

Helicases play a crucial role in many cellular processes such as DNA replication, transcription, translation, recombination, DNA repair and ribosome biogenesis. Most helicases translocate on nucleic acids powered by ATP hydrolysis, and the translocation often leads to the separation of the duplex DNA or RNA, resulting in the restructuring of DNA or RNA. The translocation and/or unwinding by several DNA helicases such as Rep, PcrA, UvrD, RecBCD and XPD, and a few RNA helicases such as NS3, have been extensively studied by both ensemble and single-molecule approaches (1–6). Interestingly, recent studies unveiled that some helicases translocate on or unwind nucleic acids repetitively by moving backward to the original binding position without dissociation. Rep and PcrA translocate repeatedly on single strand (ss) DNA, albeit with different mechanisms (1,2). XPD (ERCC2) helicase unwinds double strand (ds) DNA in a repetitive manner (5). PcrA-like helicase FBH1 undergoes repetitive motion on ss–dsDNA junction (7). NS3 also showed a repetitive unwinding of dsDNA, but only when the duplex end was blocked (6). RIG-I translocates repetitively along dsRNA without unwinding it (8). These reports suggest that the repetitive translocation or unwinding activity of helicases could be a shared molecular mechanism for some family of helicases, but how the repetitive translocation/unwinding can contribute to the biological function is still unclear.

RNA helicase A (RHA or DHX9) is a DExH-box helicase, a member of Superfamily (SF) 2. This protein is essential for mammalian embryogenesis (9) and has been shown to participate in diverse cellular functions including transcription (10), translation (11), RNA interference pathway (12) and innate immune response (13). RHA can promote replication of a number of viruses including HIV-1 (14–18). During transcriptional regulation, RHA has been shown to mediate the association of the CREB-binding protein (CBP) or BRCA1 with RNA polymerase II (19,20), and to interact with DNA and topoisomerase II-alpha (21).

RHA consists of two double stranded RNA binding domains (dsRBDs) at its N-terminus and a RGG box at C-terminus, both of which have been suggested to regulate its helicase activity (22), but not much is known about the molecular mechanism. To shed light on the role of RHA, the molecular mechanism involved in RNA unwinding by RHA was investigated using single-molecule Förster Resonance Energy Transfer (smFRET) (23), a technique that enables detection of unwinding by a single RHA on a single duplex RNA molecule in real-time. Our results revealed three interesting and unique features in RHA unwinding process. First, the unwinding of duplex RNA is preceded by a period of activation which is under the control of the N-terminal dsRBDs. Second, a single RHA molecule unwinds small regions within the dsRNA repeatedly, without dissociation of the RHA, until complete denaturation of the RNA duplex is achieved. Third, the repetitive unwinding promotes enhanced annealing of a complementary ssRNA.

## MATERIALS AND METHODS

### Cell culture

HEK 293E cells are a stably transfected HEK 293 cell line that constitutively expresses the Epstein-Barr virus nuclear antigen 1 (EBNA1) (24) and were obtained from Yves Durocher (Biotechnology Research Institute, Montreal). This cell line was adapted to grow in suspension in F17 medium (Invitrogen) supplemented by 2 mM l-glutamine and 0.1% Pluronic F-68 (Gibco), and transfected by using 25 kDa linear polyethylenimine (PEI, pH 7.0) (Polysciences Inc). EBNA1 promotes amplification of plasmid containing the replication origin region (OriP) of Epstein-Barr virus, leading to high expression of proteins encoded for by these plasmids.

### Purification of protein from 293E cells

Purification and characterization of wild-type RHA has been described previously (25). We follow the same procedure to purify 6×His tagged mutant ΔdsRBDs-RHA containing deletion of both dsRBD1 and dsRBD2 (from amino acids 2–250). Plasmid pRHA_?dsRBD1-2_ encoding ΔdsRBDs-RHA was constructed (26) and transfected into HEK 293E cells. Forty-eight hours later, transfected cells were collected, washed with ice-cold phosphate-buffered saline and then lysed in lysis buffer (50 mM NaH_2_PO_4_, 300 mM NaCl, 10 mM imidazole, 0.5% Triton X-100, 10% glycerol, protease inhibitor cocktail tablets (Roche), pH 7.4). Cell lysates were cleared by centrifugation and then incubated with Ni-nitrilotriacetic acid (NTA) agarose (Qiagen) at 4°C for 2 h to capture His-tagged proteins. After extensive washing, recombinant proteins were eluted by 250 mM imidazole solution (pH 7.4). Glutathione S-transferase (GST) was isolated from HEK 293E cells as described previously (26). The purified proteins were dialyzed against dialysis buffer (25 mM HEPES, pH 7.5; 200 mM NaCl; 10% glycerol; 4 mM DTT) and then stored at −80°C. The purity and the identity of purified protein were determined by Coomassie Brilliant Blue R250 staining and western blot analysis using anti-His, respectively. Bio-Rad protein assay reagent was used to determine protein concentration.

### RNA labeling by fluorescein-5-thiosemicarbazide

The 3'-terminus of oligonucleotides C30-G18 and C48 was labeled with fluorescein-5-thiosemicarbazide (FTSC) as described previously (27) with minor modification. Briefly, the oligonucleotides were incubated in a volume of 200 μl of 2.5 mM NaIO_4_ and 100 mM NaOAc (pH 5.0) for 1 h on ice and then precipitated with one volume of 2-propanol. After washing with 70% ethanol, the RNA was incubated in 200 μl of 100 mM NaOAc (pH 5.0) and 0.5 mM FTSC on ice overnight. The FTSC-labeled RNA was precipitated in ethanol, dissolved in 25 mM Tris–HCl, pH 7.5, and further purified by using Microspin G-25 columns (GE Healthcare).

### Fluorescence polarization assay

Briefly, 20 μl reaction mixtures containing 10 nM FTSC-labeled RNA and the indicated amounts of protein in binding buffer (10 mM Tris–HCl, pH 8.0, 50 mM KCl, 2 mM MgCl_2_, 2 mM dithiothreitol, 2.5 mM NaH_2_PO_4_, 15 mM NaCl, 4% glycerol) were incubated in 96-well Black PS HE microplate (Molecular Devices) for half an hour. Fluorescence polarization (FP) assay were then carried out in quadruplicate on a Synergy 4 multi-mode microplate reader (BioTek) to measure the polarization change. The equilibrium dissociation constant (*K*_d_) was obtained by fitting the binding curves using KaleidaGraph (28).

### Oligonucleotide labeling by Cy5 or Cy3 and annealing

DNA and RNA oligonucleotides were purchased from IDT or Dharmacon, and Cy3 and Cy5 NHS ester from GE Healthcare. For a dye labeling of oligonucleotides, excess dyes were mixed together with DNA or RNA in 100 mM NaHCO_3_, pH 8.5 and incubated overnight, and then residual dyes were removed by Biorad P-6 column or ethanol precipitation. Labeling efficiency was calculated by measuring UV–VIS spectrum, which was >90% for all labeling reactions. To generate substrate duplexes, a labeled or non-labeled ssDNA or ssRNA was mixed with a desired complementary one in an annealing buffer (10 mM Tris, pH8.0 and 100 mM NaCl), heated at 90°C for 2 min, and slowly cooled down to room temperature.

### Single-molecule FRET assays

We monitored the unwinding reaction of RHA in real-time using single-molecule FRET by a home-made wide-field total internal reflection fluorescence (TIRF) microscopy. Fluorophore-labeled oligonucleotide, a substrate of RHA, was immobilized on a PEG-coated quartz surface for single-molecule FRET assays. The emission from single fluorophores by an excitation of a solid-state 532 nm laser (Spectra Physics) is collected through a water-immersion Olympus objective (60×, NA = 1.2). Then it passes through 555 nm long pass filter which rejects Rayleigh scattering of the laser, and a dichroic mirror (cutoff: 630 nm) which separates the emission by two, a green (*I*_D_) and red emission (*I*_A_). The separated emission is detected by an Electron Multiplying Charge Coupled Device (EMCCD) camera (Andor) with a 100-ms exposure time, which is analyzed using a custom-written IDL and MATLAB program. FRET is calculated by *I*_A_/(*I*_D_ + *I*_A_), and total intensity by *I*_D_ + *I*_A_. FRET histograms were obtained by analyzing initial 10 frames of FRET traces using >5000 molecules, and FRET traces by analyzing each single molecule in real-time.

All the unwinding experiments were done in the unwinding buffer (20 mM Tris, pH 7.5, 25 mM NaCl, 3 mM MgCl_2_, 1 mM DTT (Dithiothreitol) and 0.1 mg/ml BSA (Bovine Serum Albumin)) with various concentration of RHA (10–80 nM for a wild-type RHA and 40–320 nM for ΔdsRBDs-RHA) and ATP (10 μM–1 mM) in combination with 5–10 mM trolox and the oxygen scavenger system (0.5% (w/v) glucose, 100 mg/ml glucose oxidase (Sigma) and 8.8 kU/ml catalase (Calbiochem)), which stabilize the fluorophore during a single-molecule data acquisition.

## RESULTS

### Real-time monitoring of RNA unwinding process by RHA

**Figure 1.**
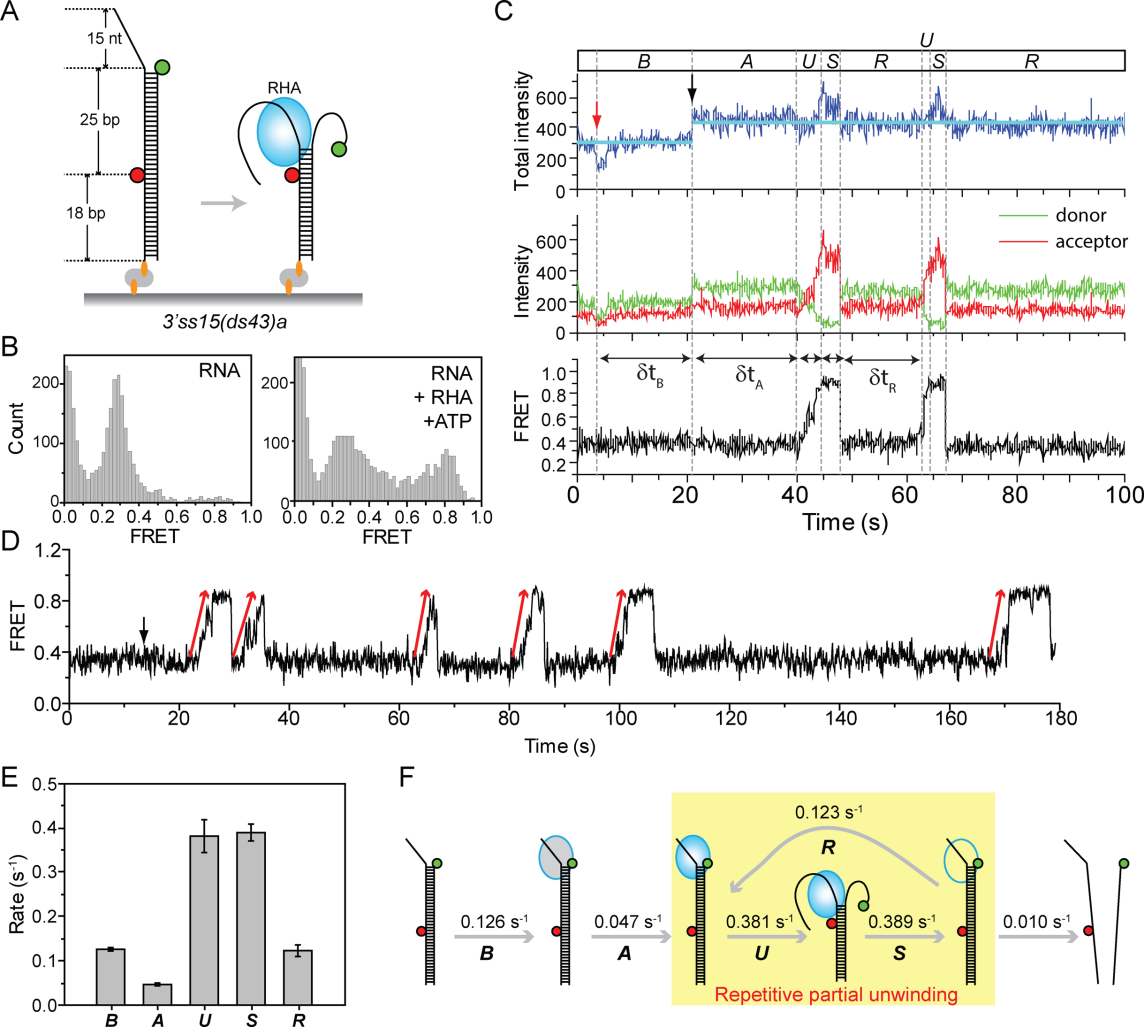
RHA unwinds dsRNA stepwise in a repetitive manner. (**A**) A diagram of an immobilized RNA substrate used for unwinding. (**B**) FRET histograms showing the RNA unwinding of RHA in the presence of ATP. (**C**) A representative smFRET trace showing the real-time RNA unwinding process of RHA, which can be dissected into five distinct kinetic substeps (*B* for *binding*, *A* for *activation*, *U* for *unwinding*, *S* for *stalling* and *R* for *re-activation*). The red arrow indicates the moment of adding RHA and ATP. The black arrow indicates the initial RHA binding to RNA. (**D**) A representative smFRET trace showing multiple rounds of unwinding upon binding of a single RHA to an RNA molecule. (**E**) The kinetic rate of each substep determined in the presence of 40 nM RHA and 1 mM ATP at room temperature. All error bars denote SEM from at least three independent experiments. (**F**) Proposed model of unwinding mechanism of RHA.

RHA unwinds dsRNA from 3′ to 5′ (Supplementary Figure S1). To monitor the mechanistic details involved in RNA unwinding process, we designed an unwinding substrate with 3′ ssRNA tail. A Cy3 (green)-labeled strand was annealed to a Cy5 (red)-labeled complementary strand that is also biotinylated. The annealed substrate is immobilized to a PEG-coated quartz surface via a biotin-NeutrAvidin linkage for total internal reflection fluorescence imaging (29). Due to the dye to dye distance (25 bp), this RNA yields low FRET by itself, and a FRET increase can be expected only when unwinding occurs as the coiling of the unwound ssRNA brings the two dyes closer to each other (Figure 1A).

The FRET histograms built from over 5000 single molecule traces obtained with the RNA substrate shows low FRET peak expected from the separation between the two dyes (Figure 1B). The FRET remained unchanged when RHA (40 nM) alone was added, suggesting that RHA binding does not disrupt the duplex significantly (Supplementary Figure S2). In contrast, when RHA was added with ATP (1 mM), a population of higher FRET molecules appeared, signifying a strand separation in an ATP-dependent manner (Figure 1B). The single molecule traces revealed a repetitive nature of unwinding exhibited by RHA evidenced by successive bursts of FRET peaks (Figure 1C and D). The moment at which RHA was added is demarcated with a red arrow. RHA binding to RNA can be detected as an abrupt increase in total fluorescence intensity (Figure 1C, black arrow) since the protein binding near fluorescent dye results in an enhancement of the fluorescence (30). Therefore, the total intensity is a useful marker for discerning the moment of protein binding and dissociation. For instance, the trace in Figure 1C shows no decrease after RHA binding, suggesting a single RHA activity without dissociation. We took advantage of this total intensity to calculate the off rate as shown below.

There are two interesting features of RHA unwinding that are evident from the real-time single molecule traces. First, the unwinding involves a multi-step process, and second, this multi-step process occurs repetitively. Based on the distinct steps present in the unwinding traces, we have divided these substeps into *binding* (*B*), *activation* (*A*), *unwinding* (*U*), *stalling* (*S*) and *reactivation* (*R*) (Figure 1C). The *binding* (*B*) refers to the time delay between RHA addition and RNA binding, whereas the *activation* (*A*) indicates the lag time between the RNA binding and the start of unwinding. This is followed by the *unwinding phase* (*U*) where FRET increases gradually (shown in more detail in Supplementary Figure S3), *stalling state* (*S*) where high FRET remains before it rapidly decreases, and the *reactivation period* (*R*) which is the second lag time before RHA initiates the next unwinding cycle. The decrease in FRET after the unwinding is interpreted as the re-zipping or rewinding of the dsRNA as RHA moves back to its initial binding site. The FRET decrease is not due to the dissociation of RHA since the total intensity remains at the same level (Figure 1C). This multi-step unwinding cycle is repeated multiple times before the two RNA strands are completely separated from each other, and the RHA dissociates (Figure 1D and Supplementary Figure S4). To address whether this repetitive unwinding is an intrinsic property of RHA or an artifact arising from specific substrate design, we compared three alternate substrate arrangement. RHA exhibited the repetitive unwinding in all three cases (Supplementary Figure S5A and B), indicating that the repetitive unwinding is likely an intrinsic behavior of RHA rather than induced by a fluorophore position or immobilization of RNA. The number of repetitive unwinding ranges between 2 and 10 cycles, likely underestimated due to the photobleaching of dyes. The number of repetition may be reduced for shorter length or weakly structured RNA that may be unwound easily.

The transition rates were calculated by analyzing the dwell times collected from over one hundred unwinding events (Figure 1E and supplementary Figure S6), and show that the *activation state* (*A*) is likely the main rate limiting step in the RHA unwinding reaction. Based on these observations, we propose a mechanism of RHA unwinding in which the successive unwinding that involves slipping back of RHA followed by a *reactivation step* in preparation for new unwinding. This cycle is repeated multiple times before the two RNA strands are completely separated from each other, and the RHA dissociates (Figure 1F).

### Kinetic analysis of the substeps in dsRNA unwinding by RHA

**Figure 2.**
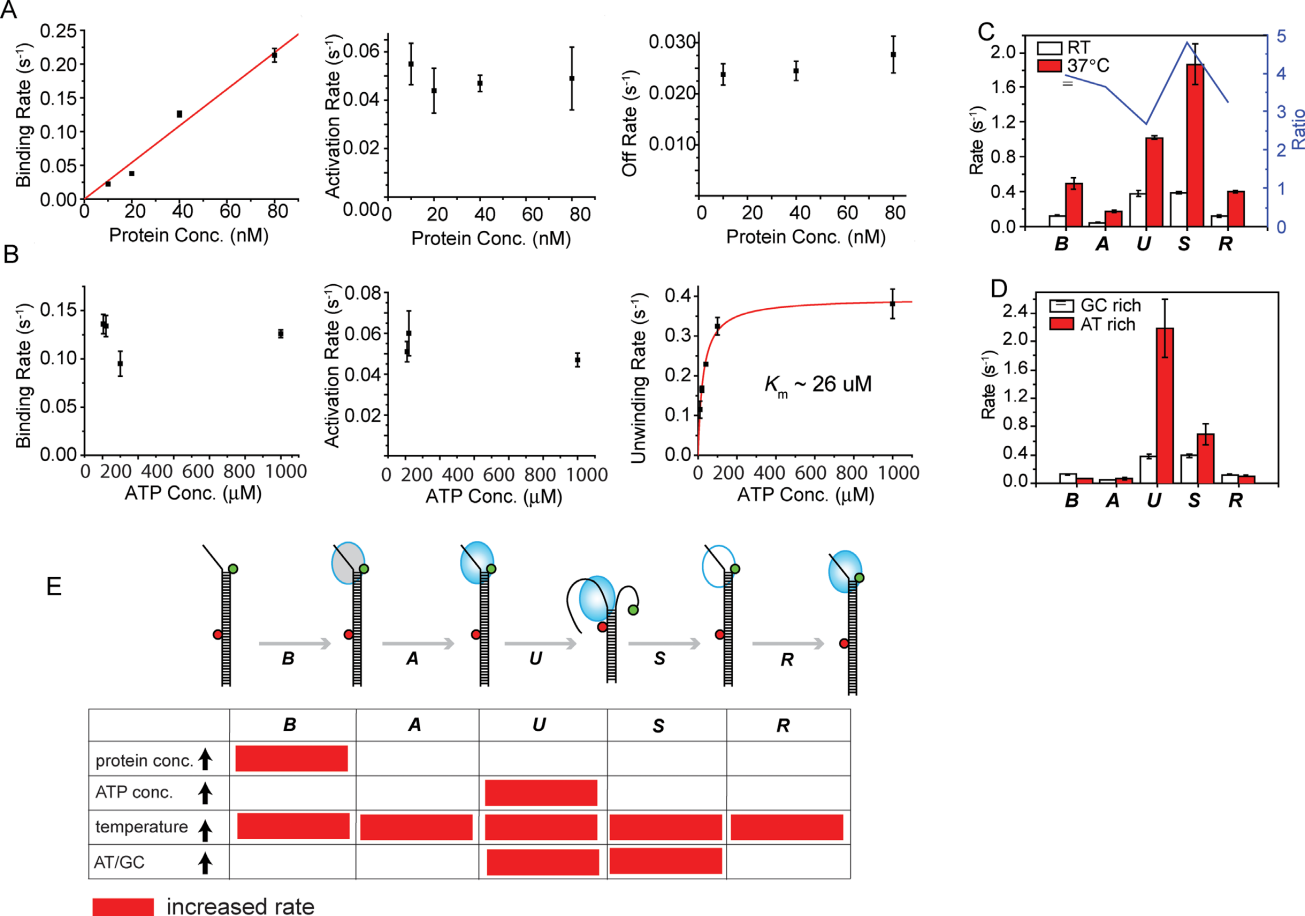
Kinetic characterization of each substep in the RNA unwinding process of RHA by varying RHA or ATP concentrations, temperature or duplex sequence composition. (**A**) The rate of RHA binding to dsRNA increases linearly with the increasing RHA concentration, giving an association constant of 2.61 μM^−1^ s ^−1^ (left). RHA concentration does not affect the activation of RHA (middle) and the off rate of RHA (right). (**B**) ATP concentration does not change binding (left) and activation rate (middle), yet it modulates unwinding rate (right). The *K*_m_ is ∼26 μM from a Michaelis–Menten plot (right). (**C**) RHA unwinding at 37°C displaying accelerated rates for all the substeps in the unwinding process. The ratio of the kinetic rate at 37°C to the one at RT is displayed as a blue line with a right *y*-axis. (**D**) AT-rich RNA sequence gives rise to a dramatic enhancement of the *unwinding* substep, a slight increase in the *stalling* rate, but does not significantly affect other substeps. All error bars denote SEM from at least three independent experiments.

Next, we investigated how the kinetic rates of the five distinguishable steps in unwinding (*B*, *A*, *U*, *S*, *R*) were affected by varying the RHA concentrations, ATP concentrations, temperature, and dsRNA sequences. As expected, the binding (*B*) rate increased linearly with increasing RHA concentration, yielding an association constant of 2.61 × 10^6^ M^−1^ s^−1^ (Figure 2A). In contrast, the activation rate and the off rate (calculated from the total bound time) showed no dependence on RHA concentration (Figure 2A). This further supports the interpretation that a single RHA molecule is responsible for the repetitive unwinding rather than successive binding of multiple RHA molecules.

When ATP concentration was changed, neither the binding (*B*) nor activation (*A*) rates were altered (Figure 2B). However, a clear ATP dependence was observed for unwinding (Supplementary Figure S7A), yielding *K*_m_ of 26 μM ATP from Michaelis–Menten fitting (Figure 2B), which is consistent with a previous report (31). At 37°C, the rates of all the substeps were increased by 2–5-fold compared to the rates at 22°C, possibly due to the faster rate of conformational change, ATP hydrolysis and RHA diffusion at the higher temperature (Figure 2C and Supplementary Figure S7B). When two different dsRNA sequences, GC-rich (60% GC) or AT-rich (40% GC), were tested, the unwinding (*U*) and the stalling rate (*S*) showed the largest difference among all the substeps, likely due to the higher thermal stability of the GC-rich strand (Figure 2D). Taken together, we show that the protein concentration, ATP concentration and base composition each modulates a specific substep(s) selectively whereas the temperature has a more global effect (Figure 2E).

### The activation step is regulated by dsRBDs

**Figure 3.**
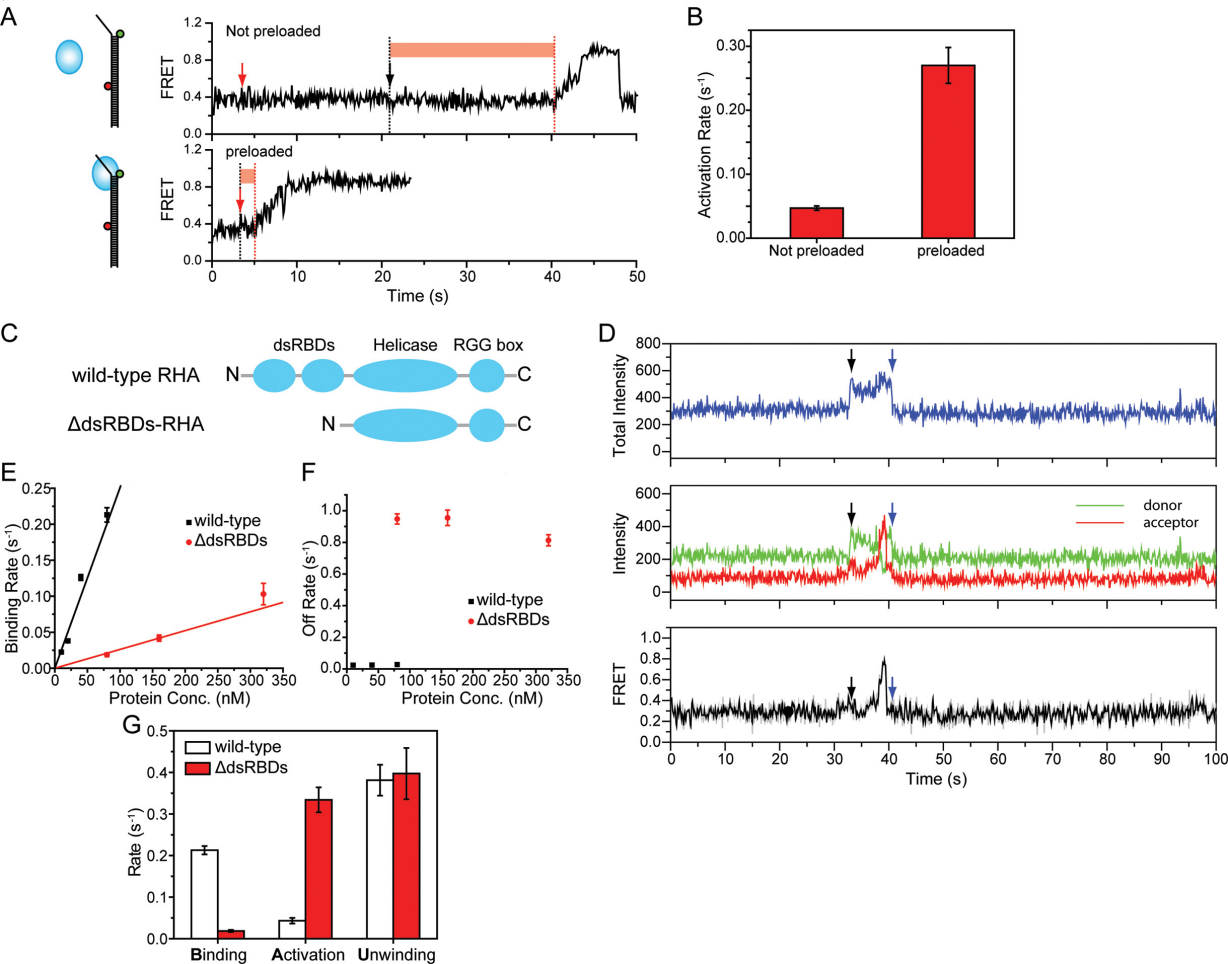
The activation of RHA for RNA unwinding is enhanced by RHA preloading, but is negatively regulated by its dsRBDs. (**A** and **B**) A representative smFRET trace of RNA unwinding when 40 nM RHA and 1 mM ATP were added together to free RNA (top) or when 1 mM ATP was added to RHA-preloaded RNA (bottom). Red arrow marks the moment of adding reagents. Black arrow marks the moment of RHA binding to RNA. RHA *activation* period is marked with a red bar and was used to calculate the *activation* rates graphically shown in (B). (**C**) Domain structures of both wild-type RHA consisting of two dsRBDs, helicase core and RGG box, and of mutant ΔdsRBDs-RHA. (**D**) Representative smFRET traces of RNA unwinding by ΔdsRBDs-RHA shows a shorter activation period preceding the RNA strand separation and one-time unwinding cycle followed by protein dissociation. The black or blue arrows indicate the moments of protein binding or dissociation, which are demonstrated by the increase or decrease of total intensity, respectively. (**E**) The binding rates for wild-type and ΔdsRBDs-RHA analyzed over varying protein concentration. (**F**) The off rate measured for the wild-type RHA and ΔdsRBDs-RHA. (**G**) The rates collected for substeps of the wild-type and ΔdsRBDs-RHA.

To characterize the activation step in the RNA unwinding, we tested whether preloading RHA would accelerate the activation step. When unwinding reaction was initiated with (bottom) or without (top) preloading RHA, the activation rate was considerably increased in the preloaded case, suggesting that the activation step involves a proper loading of RHA to dsRNA (Figure 3 A and B). This result also suggests that the proper RHA loading to dsRNA does not require ATP, as the preloading was performed in the absence of ATP.

Since N-terminal dsRBDs are thought to be essential for RHA to target RNA substrate (26), we tested the role of this domain in RNA loading and unwinding activity by preparing dsRBD truncation mutant (ΔdsRBDs-RHA) (Figure 3C and D). We found that the ΔdsRBDs-RHA exhibited lower binding rate than the wild-type RHA (Figure 3E), which is coupled with a higher off rate in the 10–320 nM protein concentration range (Figure 3F). The high off rate of ΔdsRBDs-RHA might be responsible for the lack of repetitive unwinding in this mutant (Figure 3D and Supplementary Figure S8A), indicating that dsRBDs is causally associated with the repetitive unwinding behavior of wild-type RHA. This suggests that the dsRBDs contribute to both the increased binding affinity as well as the stability of RHA binding to dsRNA (Figure 3E and F). We confirmed the affinity effect by the fluorescence polarization measurement (Supplementary Figure S8B).

The analysis of individual rates showed that while the binding rate is decreased, the activation rate was increased in the ΔdsRBDs-RHA and the unwinding rate remained unchanged (Figure 3G). This suggests an inhibitory role of dsRBDs for the activation, but not for unwinding step. When the inhibitory dsRBD is removed, the ΔdsRBDs-RHA initiates unwinding readily. Further, the activation rate measured for ΔdsRBDs-RHA is similar to the enhanced activation rate of preloaded wild-type RHA (Figure 3B and G), indicating the dsRBDs as the primary factor responsible for the slower activation step in the wild-type RHA. Taken together, the dsRBDs increase the binding affinity of RHA to dsRNA and stabilize its binding while negatively regulating its activation for unwinding.

### Repetitive unwinding assists annealing activity of RHA

**Figure 4.**
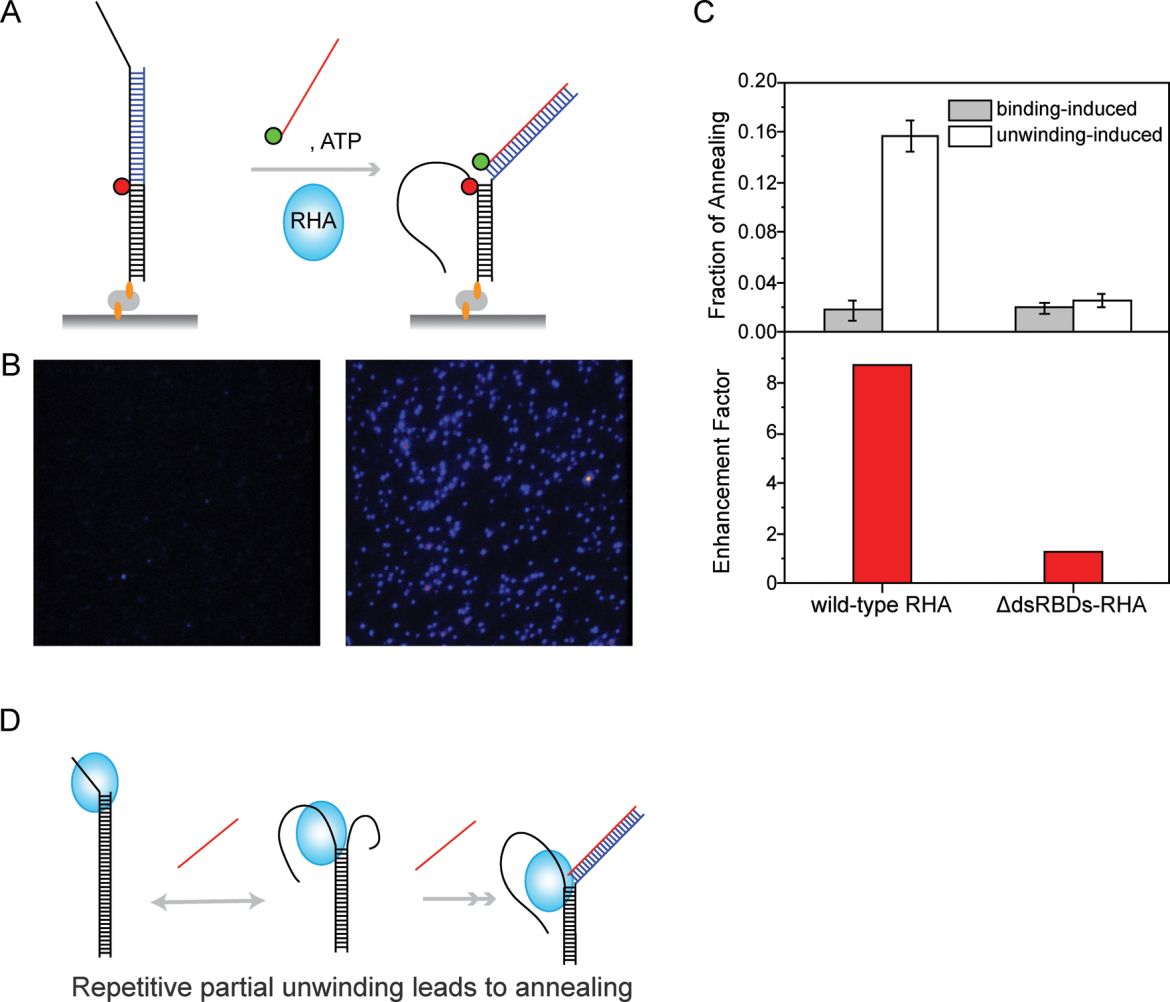
Repetitive RHA unwinding enhances the annealing of ssRNA to its complementary strand in dsRNA. (**A**) An experimental scheme for testing the annealing activity of RHA. The RNA strand (blue line) is complementary to the Cy3-labeled ssRNA (red line). (**B**) Single molecule surface images taken with (right) and without (left) RHA and ATP. (**C**) The binding-induced annealing and unwinding-mediated annealing measured for wild-type and ΔdsRBDs-RHA (top) and the enhancement factor for both proteins (bottom). (**D**) A diagram showing how repetitive unwinding enhances the annealing of ssRNA to its complementary strand.

Unwinding activity of RHA has been proposed to play a critical role in remodeling of complicated RNA secondary structures (11,18,32,33) in which both disruption and formation of double-stranded RNA occur. RHA has also been shown to promote the annealing of HIV-1 genomic RNA to tRNA^Lys3^, the primer for reverse transcriptase in HIV (32). We therefore investigated whether the repetitive unwinding activity of RHA can assist annealing of ssRNA to dsRNA. We prepared Cy3-labeled ssRNA complementary to a Cy5-labeled dsRNA such that the annealing would be detected as an appearance of high FRET (Figure 4A). The ssRNA was designed to constitute a blunt-end duplex when annealed, to avoid the unwinding of the newly annealed duplex. In this platform, the appearance of high FRET molecules can be tabulated over time to estimate the rate of annealing (Figure 4B). When the Cy3-ssRNA was added to dsRNA in the absence of RHA and ATP, we observed no apparent annealing since the spontaneous melting of dsRNA is not expected in this condition. The addition of ssRNA and RHA without ATP resulted in some annealing, likely due to partial melting of duplex caused by RHA binding (Figure 4C, gray bar, binding-induced annealing). The most efficient annealing was achieved when ssRNA was added with RHA and ATP, suggesting that ATP-stimulated RHA unwinding facilitates the annealing process (Figure 4C, white bar, unwinding-induced annealing).

To test if the repetitive nature of unwinding can facilitate the annealing, we compared the annealing efficiency between the wild-type RHA and the ΔdsRBDs-RHA which typically unwinds only once (Figures 3D and 4C). To account for the difference of binding affinity between the wild-type RHA and ΔdsRBDs-RHA, we used higher protein concentration for ΔdsRBDs-RHA (320 nM) than for wild-type RHA (40 nM). The wild-type RHA with a stronger RNA binding affinity showed a higher binding-induced annealing than ΔdsRBDs-RHA even at a lower concentration (Figure 4C). In addition, we obtained an enhancement factor which is a ratio between the unwinding-induced annealing (with ATP) and the binding-induced annealing (without ATP). The comparison of these ratios showed a sufficiently larger enhancement factor than the ΔdsRBDs-RHA (Figure 4C), indicating that the repetitive unwinding of RHA facilitates annealing by making the unwound strand more accessible for pairing with the incoming RNA (Figure 4D).

## DISCUSSION

Our study reveals the detailed molecular mechanism underlying RHA unwinding. The single molecule fluorescence technique we employed here enables detection of RNA–protein interaction in unprecedented molecular detail. We extracted two types of signals from the single molecule traces. First, the stepwise increase and decrease in the total intensity (sum of Cy3 and Cy5) reported on the binding and dissociation of RHA, respectively. The dwell times of the protein bound state collected from hundreds of molecules were used to estimate the off rate of the protein. Additionally, the unchanging total intensity during the repetitive unwinding cycle reflected that a single unit of RHA continues to repeat its motion without dissociating. Second, the FRET signal reported on the unwinding and the rewinding of the RNA duplex, observed as a gradual FRET increase and a rapid FRET decrease, respectively. We note that this FRET arrangement is preferred over the high FRET RNA where FRET decrease reports on unwinding (34) because FRET decrease can be indistinguishable from photobleaching of a fluorophore. Using both the total intensity and FRET signals, we resolved the substeps of RHA unwinding that include RHA binding (*B*), activation (*A*), dsRNA unwinding (*U*), stalling (*S*) and, a reactivation step (*R*). Our results indicate that the conditions such as protein and ATP concentration modulate a specific substep, while temperature influences the rate of all substeps.

**Figure 5.**
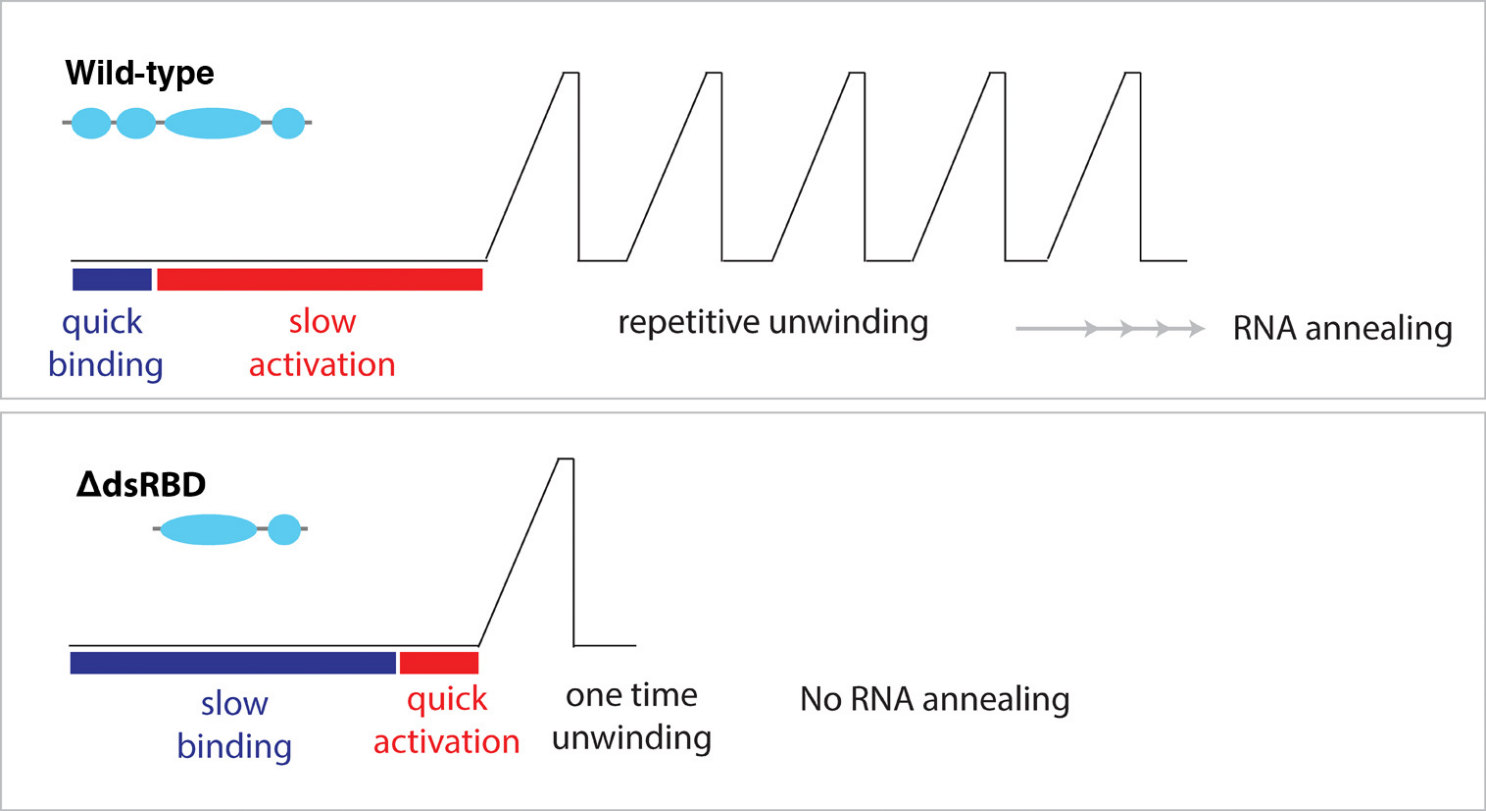
Summary. The unwinding pattern of wild-type and ΔdsRBDs-RHA is summarized. The wild-type RHA exhibits quick binding followed by slow activation and the repetitive unwinding by a single whereas ΔdsRBDs-RHA displays slow binding, fast activation followed by single unwinding. The repetitive unwinding of the wild-type leads to RNA annealing.

The unwinding characteristic of ΔdsRBDs-RHA clearly revealed the regulatory role of dsRBDs in several important aspects. It can be divided into a facilitating/promoting role versus an inhibitory effect of dsRBD. For the positive role, the dsRBDs provides a high affinity toward RNA as evidenced by a dramatic decrease in binding rate of ΔdsRBDs-RHA, even at a high concentration (Figure 3E). The dsRBDs promotes repetitive unwinding of RHA as the ΔdsRBDs-RHA shows drastically diminished repetition (Figure 3D and Supplementary Figure S5). The dsRBDs also enhances stability of RHA–RNA interaction as displayed by the high off rate of ΔdsRBDs-RHA. As a result, the dsRBDs enhances the annealing of RNA. For the inhibitory role, the dsRBDs slows down the initiation of unwinding by inducing a delay in activation (Figure 5). Our preloading experiment confirms that this lag time is required for proper loading of the protein onto the substrate. It is plausible that the strong binding of the dsRBDs restricts movement of RHA that is required for the helicase activity.

The significance of the repetitive unwinding must be considered in light of the diverse functions of RHA in the cell. RHA participates in the activation of transcription (19,20,33,35), mRNA splicing (18,36,37), and stimulation of translation (11). RHA has been found associated with the Rev/Rev-response element (RRE) complex (38), the nuclear pore complex (39) and the RNA-induced silencing complex (RISC) involved in the RNAi pathway (12). It is plausible that RHA facilitates targeting of siRNAs/miRNAs to double-stranded 3′ UTR via its repetitive unwinding activity. It is interesting to note that RHA unwinds repetitively within a certain range of RNA length, similar to the one of siRNAs/miRNAs. RHA can also act as a sensor for pathogenic dsRNA in myeloid dendritic cells, and through its interaction with IPS-1, activate the pathway leading to production of IFN-α/β and proinflammatory cytokines (40). During HIV-1 replication, RHA is also involved in promoting primer tRNA^Lys3^ annealing to viral genomic RNA, and genomic RNA dimerization (32). It is not clear whether all these functions require helicase activity, and if so, require unwinding of long stretches of RNA. In fact, some DEAD box RNA helicases unwind very short stretches (a few basepairs) of duplex without translocation (yeast protein Ded1 (41), human proteins eIF4A (42) and p68 (43), and *Escherichia coli* proteins CsdA, RhIE and SrmB, etc. (44)). These different functions of RHA may be regulated by RHA-associated cofactors found in different nucleoprotein complexes, and by internal modifications within the RHA. For example, there is an evidence that RHA and the dsRNA-dependent protein kinase, PKR, can interact with each other through their respective dsRBDs, and that PKR can phosphorylate RHAs dsRBDs, preventing association of RHA and dsRNA (45). It therefore remains to be determined if the phosphorylated RHA shows the same repetitive unwinding.

## SUPPLEMENTARY DATA

Supplementary Data are available at NAR Online.

SUPPLEMENTARY DATA
