# Supplementary material for: Repetitive RNA unwinding by RNA helicase A facilitates RNA annealing
Source: Nucleic Acids Res. 2014 Jun 9;42(13):8556–64. doi: 10.1093/nar/gku523 (PMC4117756; doi:10.1093/nar/gku523)
Supplement: SUPPLEMENTARY DATA [file supp_42_13_8556__index.html]

SUPPLEMENTARY DATA 

# Repetitive RNA unwinding by RNA helicase A facilitates RNA annealing

## SUPPLEMENTARY DATA

**Files in this Data Supplement:**

- Suplementary Information
