# Supplementary material for: Repetitive RNA unwinding by RNA helicase A facilitates RNA annealing
Source: Nucleic Acids Res. 2014 Jun 9;42(13):8556–64. doi: 10.1093/nar/gku523 (PMC4117756; doi:10.1093/nar/gku523)
Supplement: SUPPLEMENTARY DATA [file supp_gku523_nar-00940-r-2014-File007.docx]

Supplementary Information

Repetitive RNA unwinding by RNA helicase A facilitates RNA annealing

Hye Ran Koh^1,2^, Li Xing^3,4^, Lawrence Kleiman^3,4,^ Sua Myong^2,5,6^

^1^ Department of Physics,

^2^ Institute for Genomic Biology, University of Illinois, Urbana, IL61801

^3^ Lady Davis Institute for Medical Research and McGill AIDS Centre, Jewish General Hospital, Montreal, Quebec, Canada

^4^ Department of Medicine, McGill University, Montreal, Quebec, Canada

^5^ Department of Bioengineering

^6^ Physics Frontier Center (Center of Physics for Living Cells), University of Illinois, Urbana, IL61801

^7^ Biophysics and Computational Biology; 1110 W. Green St. Urbana, Illinois 61801

Correspondence should be addressed to S.M. (smyong@illinois.edu)

Supplementary Figure 1

Supplementary Figure 2

Supplementary Figure 3

Supplementary Figure 4

Supplementary Figure 5

Supplementary Figure 6

Supplementary Figure 7

Supplementary Figure 8

Supplementary Table 1


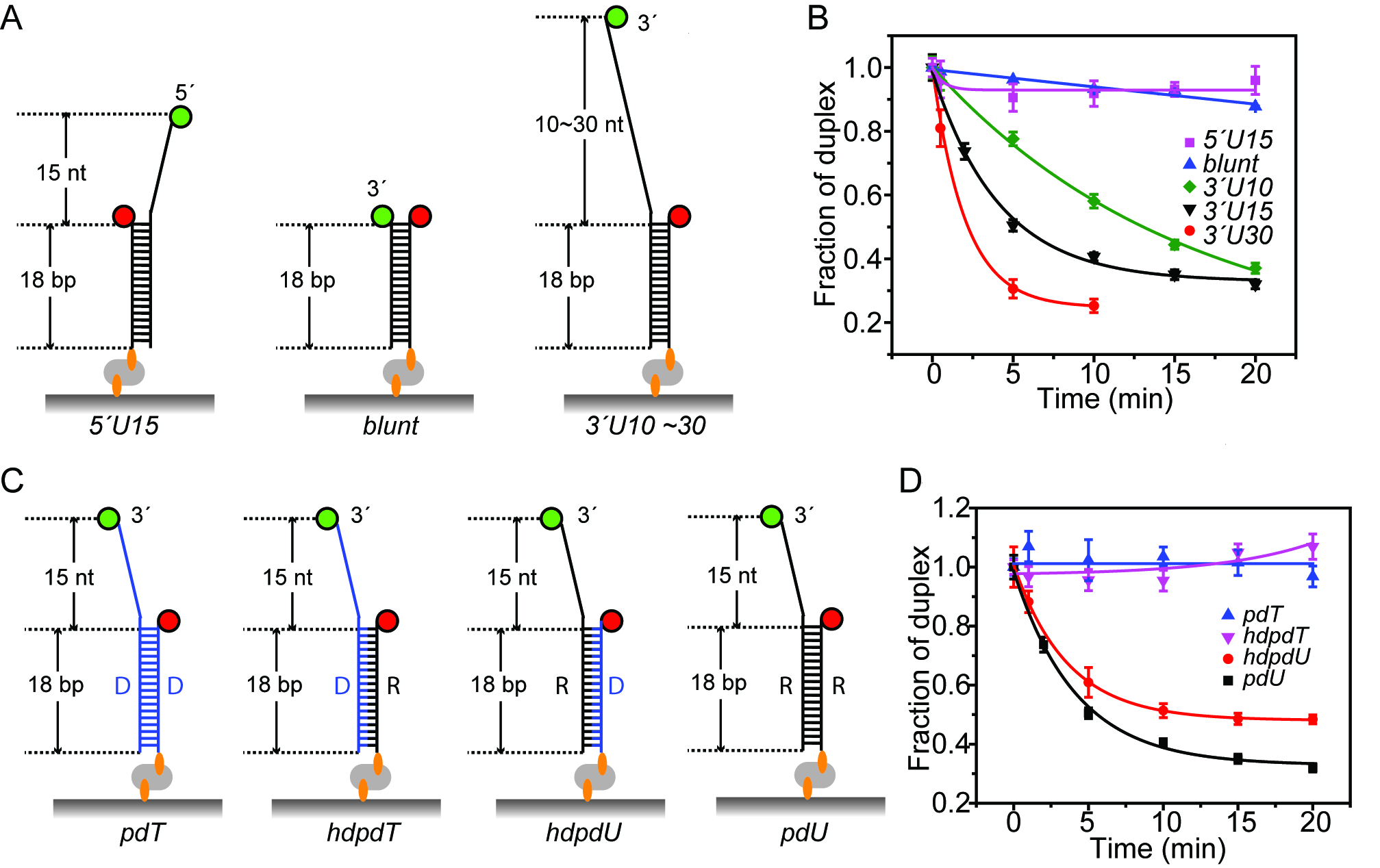


**Figure S1. RHA unwinds duplex containing 3´ ssRNA tail and RNA tracking strand.** (A) Diagrams of tested dsRNAs containing 5´ tail (*5´U15*), blunt end (*blunt*), or 3´ tail of 10 nt (*3´U10*), 15 nt (*3´U15*), or 30 nt (*3´U30*) in length. The RNA strand is labeled with Cy3 (green circle) or Cy5 (red cycle). (B) smFRET detected unwinding activity of RHA only for 3´ tailed substrates. A faster unwinding rate was observed for RNA containing a longer 3´ tail. (C) Diagrams of DNA duplex (*pdT*), DNA-RNA heteroduplex possessing DNA tracking strand (*hdpdT*), DNA-RNA heteroduplex possessing RNA tracking strand (*hdpdU*), and an RNA duplex (*pdU*). The DNA strand is in blue color, and the RNA in black color. (D) Unwinding activity of RHA was detected only for duplexes possessing RNA tracking strand. All error bars denote SEM from three independent experiments.


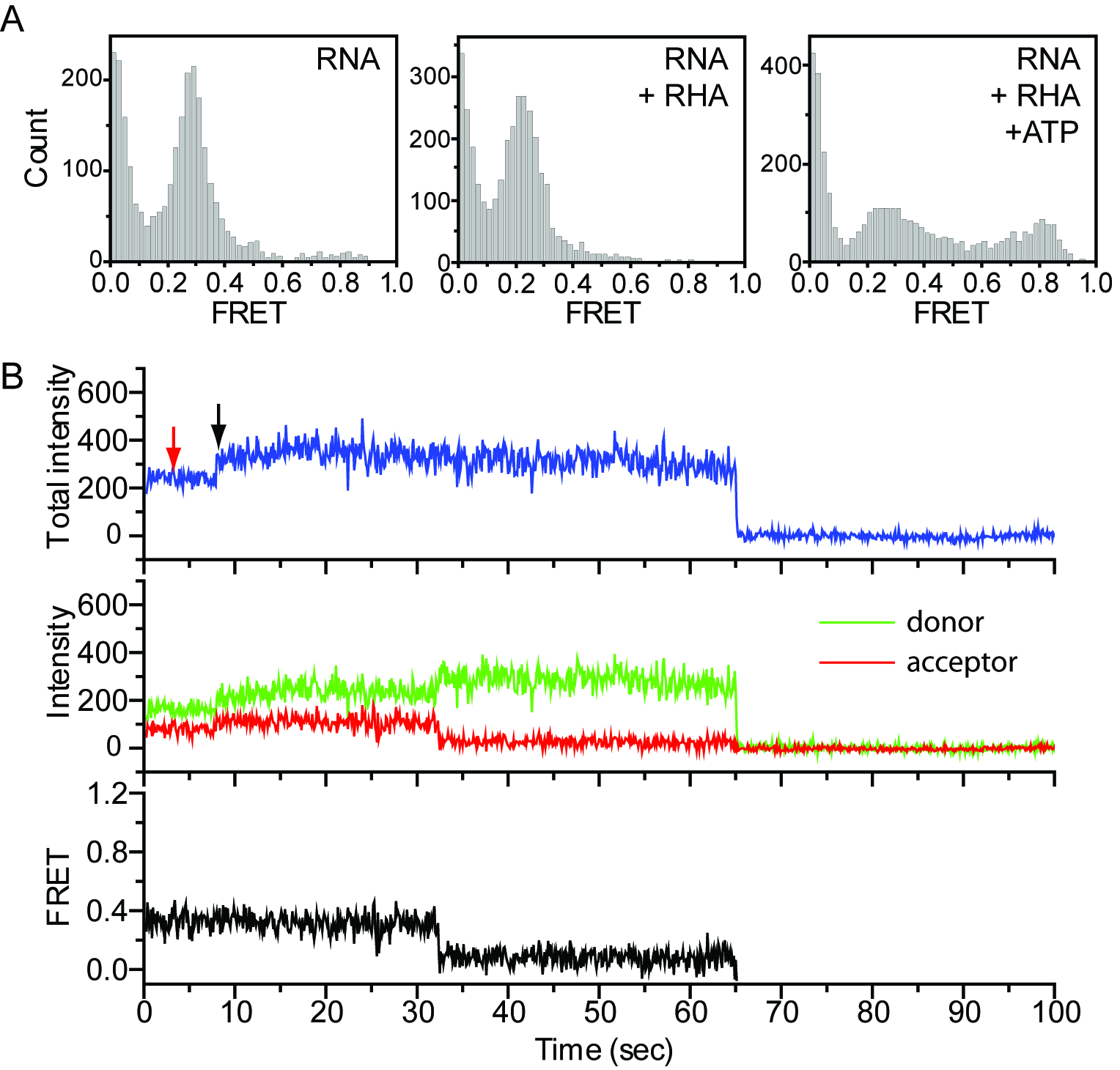


Figure S2. RHA does not show any FRET change in the absence of ATP. (A) FRET histograms showing that RHA does not show any FRET change in the absence of ATP. (B) A representative smFRET trace when RHA is added to RNA in the absence of ATP. The red arrow is marked at the moment of RHA addition and the black at the moment of RHA binding.


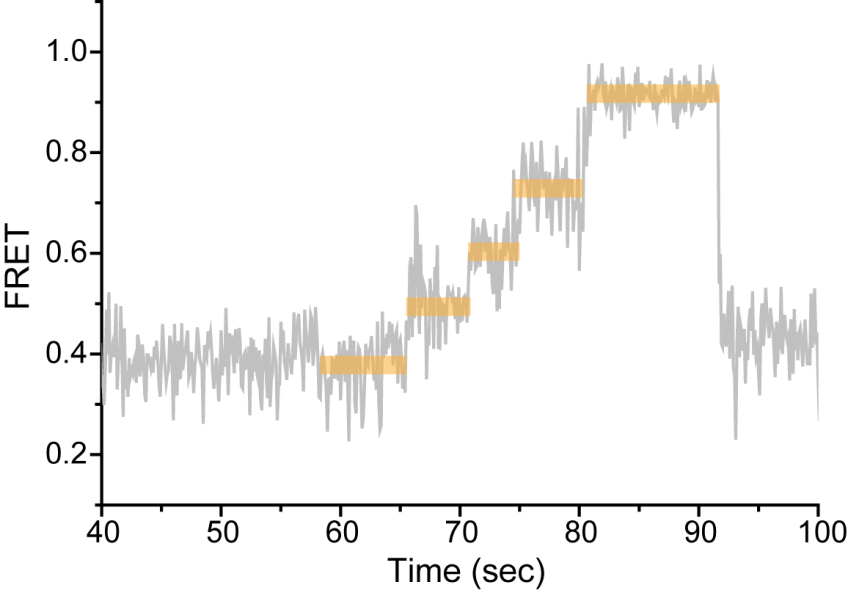


Figure S3. Real-time monitoring of the gradual FRET increase at the unwinding sub-step during RNA unwinding by RHA. A gradual increase of FRET consists of several intermediate steps (marked with orange bars) and is a signature of RNA strand separation by RHA because the FRET will not be changed until base-paring is disrupted. To get a clear view of the unwinding sub-steps, 10 uM of ATP was used to slow down the unwinding rates.


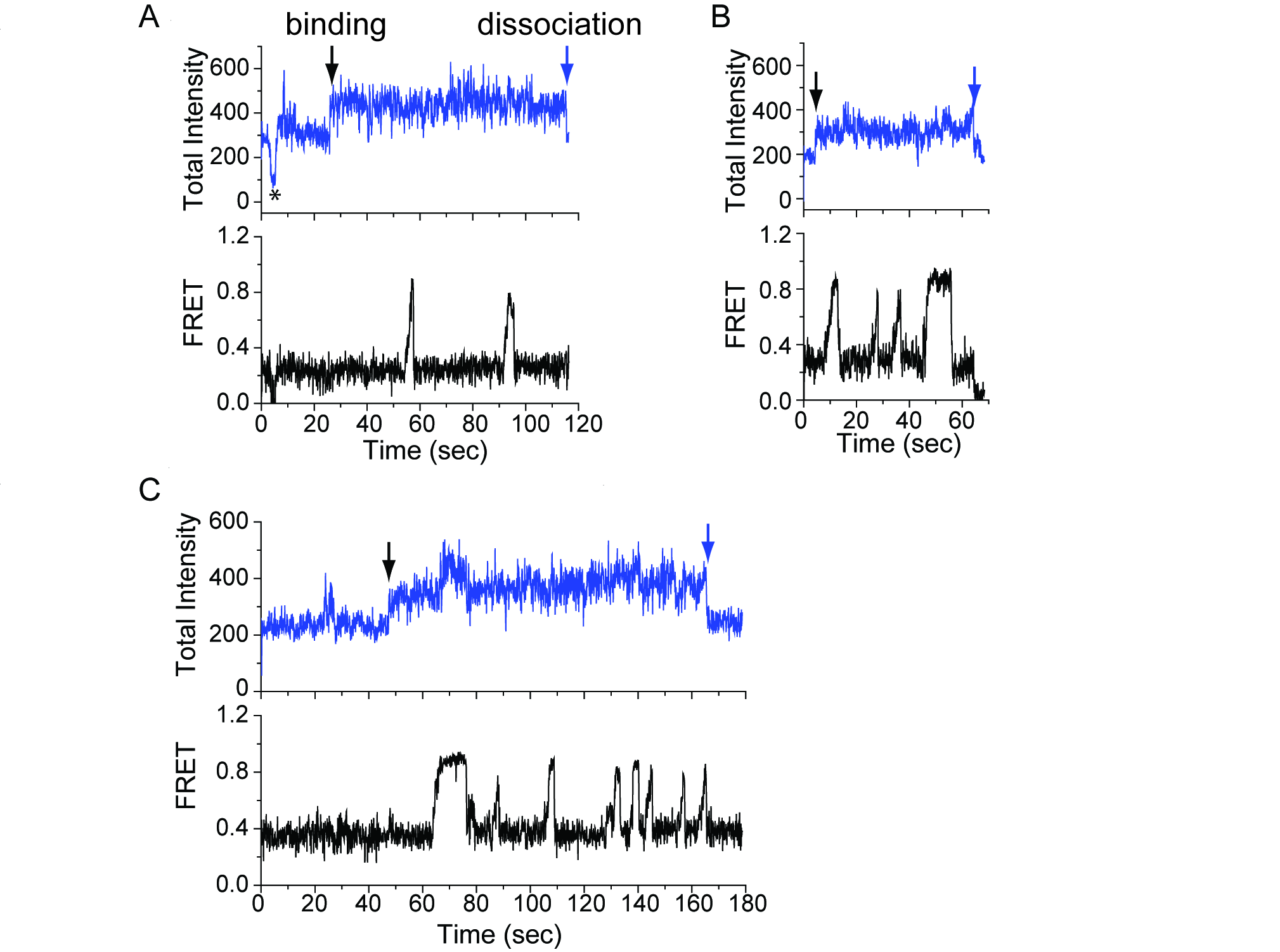


Figure S4. Representative smFRET traces of multiple unwinding cycles by wild-type RHA. Representative smFRET traces showing two (A), four (B), eight (C) unwinding cycles between RHA binding and its dissociation. The black, blue arrows indicate the moment of RHA binding and RHA dissociation, respectively.


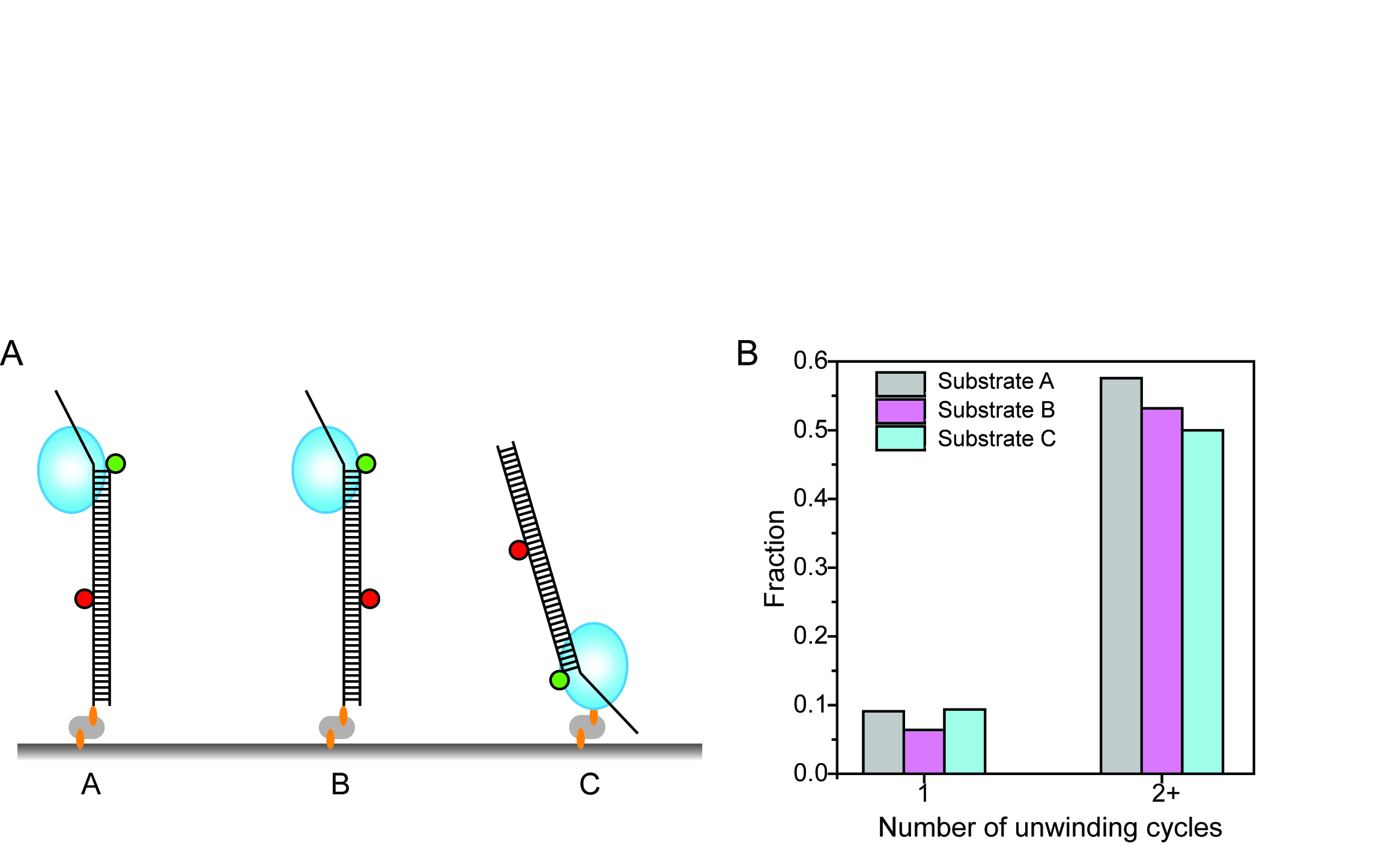


Figure S5. The repetitive unwinding is intrinsic property of RHA. (A) Three alternate substrates where the dye positions are the same as the one in Figure 1 (Substrate A), dyes are relocated to be on non-tracking strand (Substrate B) and the protein was immobilized and RNA substrate was added (Substrate C). (B)The number of single vs. multiple repetition counted for substrates A-C.


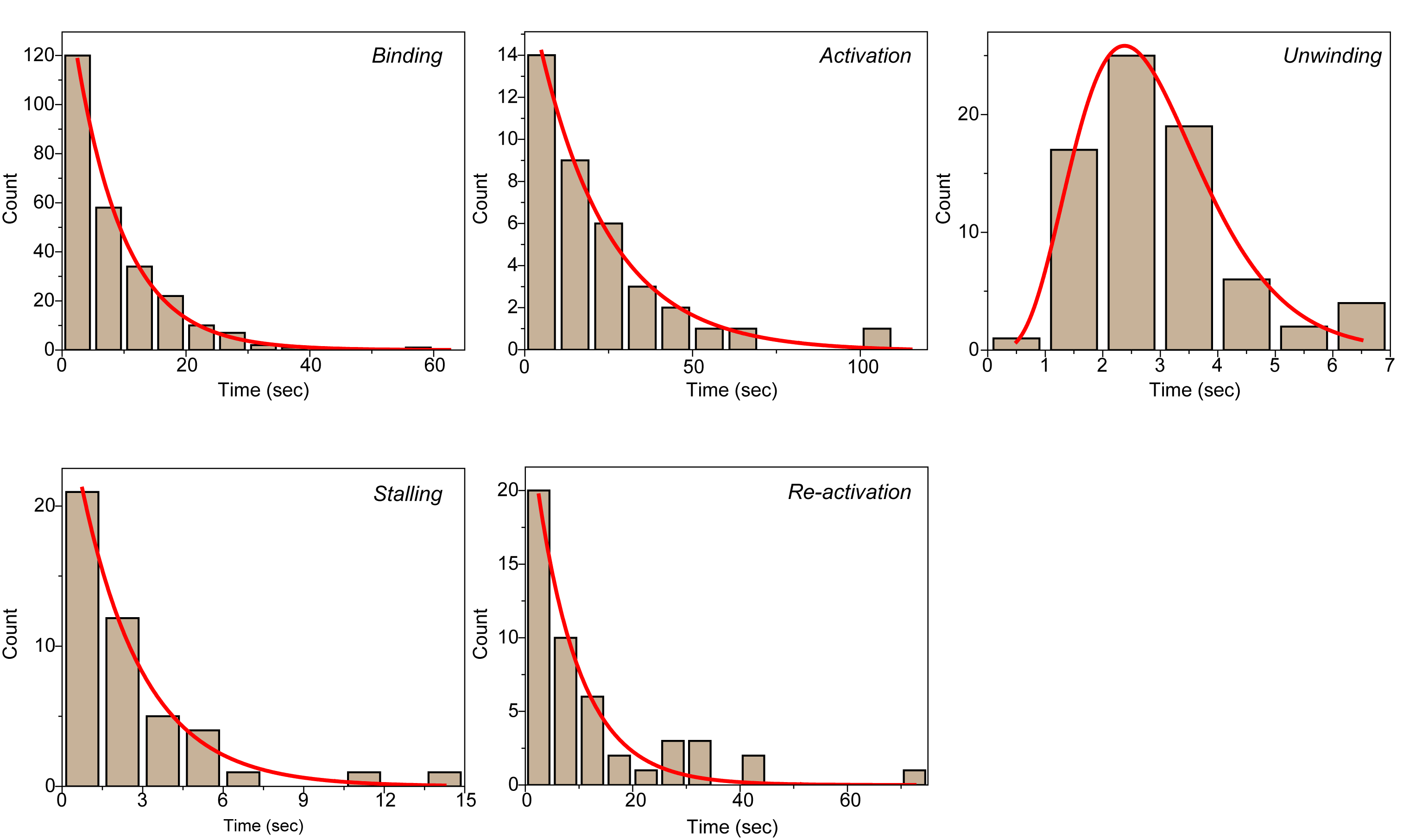


Figure S6. Dwell time distribution for each kinetic state in the RHA unwinding process. All the kinetic states except unwinding state fit to a single exponential decay, suggesting that they are single Poissonian events. The unwinding state fits to a gamma distribution, showing its characteristic of a multistep process. It matches with multiple substeps found in unwinding states as shown in Supplementary Figure S3.


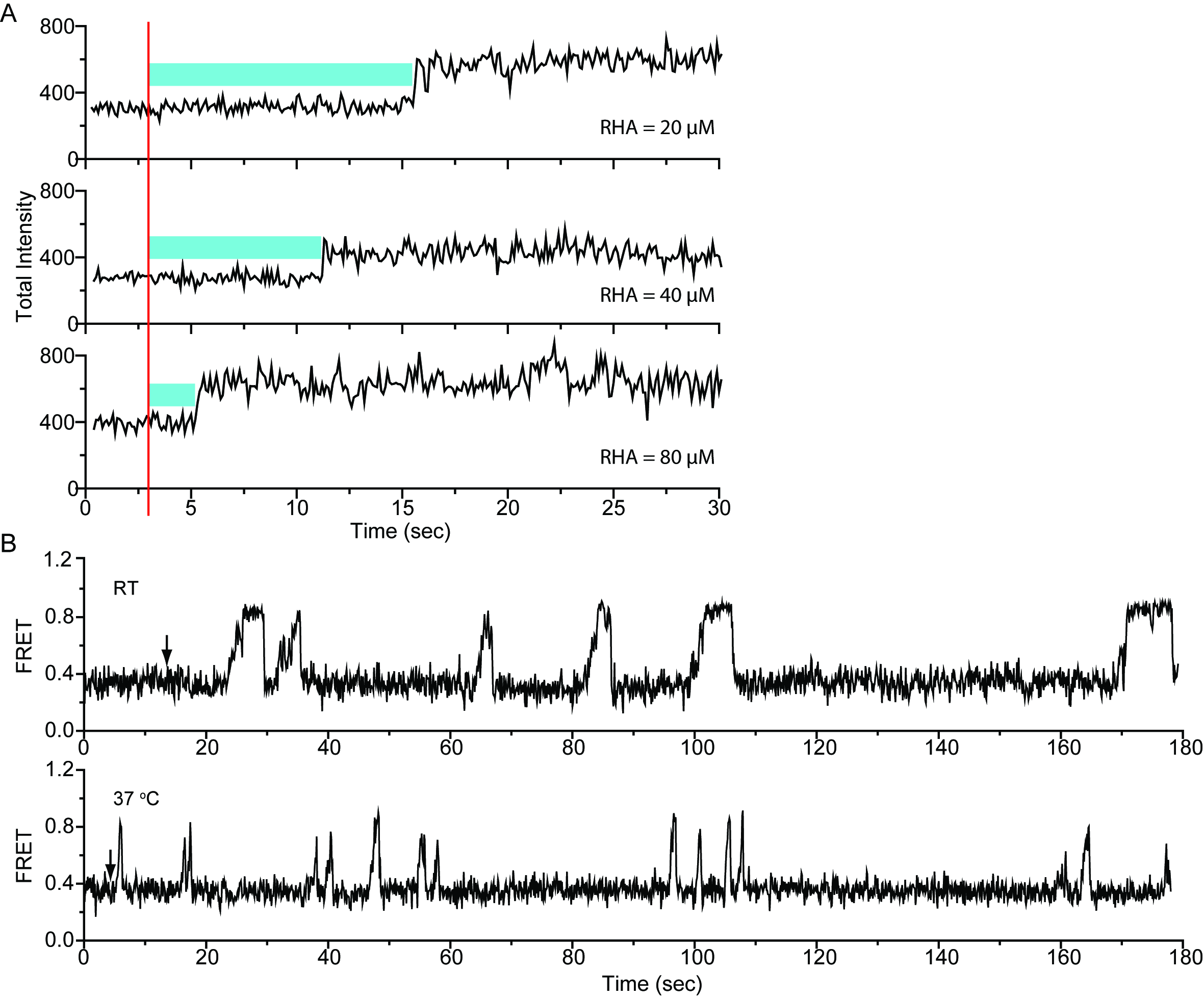


Figure S7. Representative traces of total fluorescence intensity or FRET obtained by varying RHA concentration or temperature. (A) Increasing RHA concentration shortens the time period for RHA binding sub-step, but does not affect other sub-steps during RNA unwinding by RHA. The time period for RHA binding to RNA is marked with skyblue bars. This time period starts from the red line where RHA was added and ends at the moment of increased total intensity that results from the physical binding of RHA to RNA. (B) Representative smFRET traces of RNA unwinding by RHA under room temperature (RT) and 37°C. Black arrow indicates the moment of RHA binding to RNA.


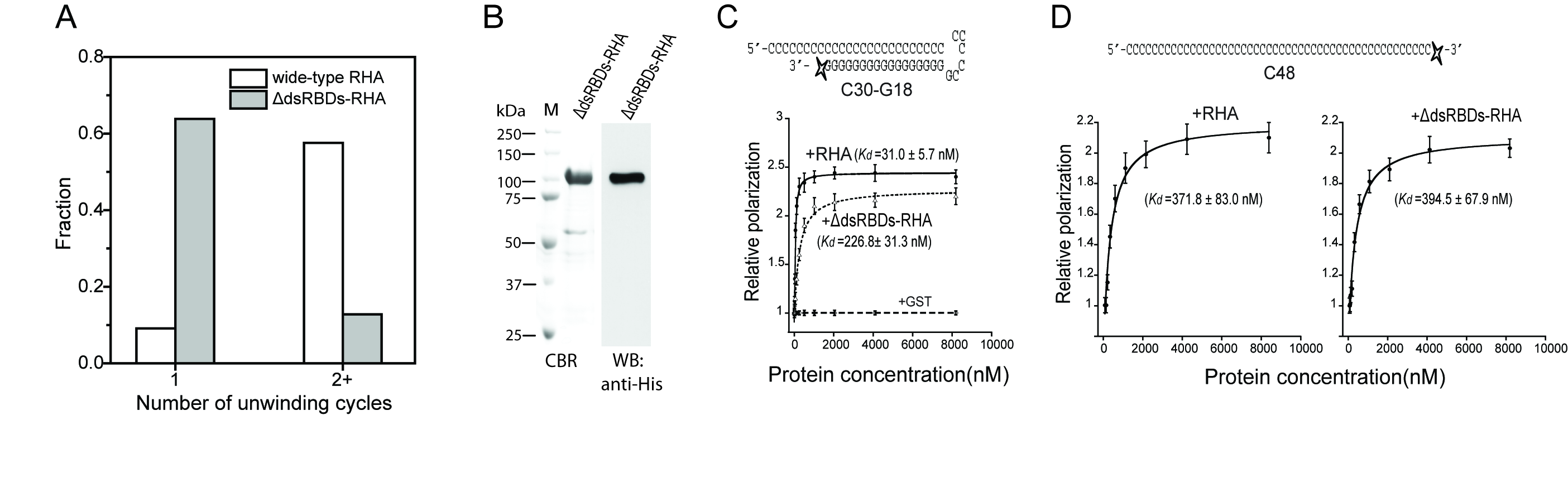


**Figure S8.** **Wild-type RHA unwinds RNA repetitively and binds to dsRNA preferentially.**

(A) Wild-type RHA not ΔdsRBDs-RHA unwinds RNA repetitively. We counted the number of unwinding cycles per RHA binding and distinguished one (1) from multiple unwinding cycles (2+) for wild-type and ΔdsRBDs-RHA. (B) Purification of 6×His tagged ∆dsRBDs-RHA containing deletion of two dsRBDs. Proteins was purified from mammalian 293E cells, separated by 1D PAGE, and analyzed by either staining with Coomassie Brilliant Blue R250 (CBR), or by Western blots probed with anti-His antibody. Lane M displays a size marker with indicated molecular weights in kDa. (C, D) Fluorescence polarization (FP) analysis of the binding affinity of wild-type RHA or ∆dsRBDs-RHA for RNA oligonucleotide C30-G18 (C) or C48 (D). Schematic representation of secondary structure of RNA is on the top of each graph. The 3′-end of C30-G18 or C48 was labeled with fluorescein-5-thiosemicarbazide (FTSC). Glutathione S-transferase (GST) was used as a non-RNA binding protein control. Apparent equilibrium dissociation constants (*K_d_*) for protein-RNA binding are shown as the mean ± standard deviation.

Table S1. The sequence of oligonucleotides used in unwinding assay of RHA.

| name | sequence |
| --- | --- |
| 5´U15 | 5´- biotin-rArCrCrGrCrUrGrCrCrGrUrCrGrCrUrCrCrG-Cy5-3´ (up)  3´- rUrGrGrCrGrArCrGrGrCrArGrCrGrArGrGrC(rU)_15_-Cy3 -5´ (down) |
| blunt | 5´- Cy5-rGrCrCrUrCrGrCrUrGrCrCrGrUrCrGrCrCrA-biotin-3´ (up)  3´- Cy3-rCrGrGrArGrCrGrArCrGrGrCrArGrCrGrGrU-5´ (down) |
| 3´U15  (pdU) | 5´- Cy5-rGrCrCrUrCrGrCrUrGrCrCrGrUrCrGrCrCrA-biotin-3´ (up)  3´- Cy3-(rU)_15_rCrGrGrArGrCrGrArCrGrGrCrArGrCrGrGrU-5´ (down) |
| 3´ U10 or 3´U30 | 5´- Cy5-rGrCrCrUrCrGrCrUrGrCrCrGrUrCrGrCrCrA-biotin-3´ (up)  3´- Cy3-(rU)_N_rCrGrGrArGrCrGrArCrGrGrCrArGrCrGrGrU-5´ (down) (N=10 or 30) |
| pdT | 5´- Cy5-GCCTCGCTGCCGTCGCCA-biotin-3´ (up)  3´-Cy3-(T)_15_CGGAGCGACGGCAGCGGT-5´ (down) |
| hdpdT | 5´- Cy5-rGrCrCrUrCrGrCrUrGrCrCrGrUrCrGrCrCrA-biotin-3´ (up)  3´-Cy3-(T)_15_CGGAGCGACGGCAGCGGT-5´ (down) |
| hdpdU | 5´- Cy5-GCCTCGCTGCCGTCGCCA-biotin-3´ (up)  3´- Cy3-(rU)_15_rCrGrGrArGrCrGrArCrGrGrCrArGrCrGrGrU-5´ (down) |
| 3´ss15(ds43)a | 5´- biotin-rUrGrGrCrGrArCrGrGrCrArGrCrGrArGrGrC-Cy5-rUrCrUrCrCrCrCrArCrCrArCrCrArUrCrArCrUrUrGrGrUrCrG(rA)_15_-3´ (up)  3´- rArCrCrGrCrUrGrCrCrGrUrCrGrCrUrCrCrGrArGrArGrGrGrGrUrGrGrUrG  rGrUrArGrUrGrArArCrCrArGrC-Cy3-5´ (down) |
